# Pro-inflammatory response ensured by LPS and Pam3CSK4 in RAW 264.7 cells did not improve a fungistatic effect on *Cryptococcus gattii* infection

**DOI:** 10.7717/peerj.10295

**Published:** 2020-11-25

**Authors:** Gabriela Yamazaki de Campos, Raquel Amorim Oliveira, Patrícia Kellen Martins Oliveira-Brito, Maria Cristina Roque-Barreira, Thiago Aparecido da Silva

**Affiliations:** Department of Cell and Molecular Biology and Pathogenic Bioagents, Ribeirão Preto Medical School, University of São Paulo, Ribeirão Preto, São Paulo, Brazil

**Keywords:** Macrophage polarization, LPS, Pam3CSK4, TLR, *Cryptococcus gattii*, RAW 264.7 cells

## Abstract

**Background:**

The macrophage lineage is characterized by plasticity due to the acquisition of distinct functional phenotypes, and two major subsets are evaluated; classical M1 activation (strong microbicidal activity) and alternative M2 activation (immunoregulatory functions). The M1 subset expresses inducible nitric oxide synthase (iNOS), which is a primary marker to identify these cells, whereas M2 macrophages are characterized by expression of Arginase-1, found in inflammatory zone 1 (Fizz1), chitinase-like molecule (Ym-1), and CD206. The micro-environmental stimuli and signals in tissues are critical in the macrophage polarization. Toll-like receptors (TLR) ligands, such as lipopolysaccharide (LPS), palmitoyl-3-cysteine-serine-lysine-4 (Pam3CSK4), and ArtinM (mannose-binding lectin) are inductors of M1 subset. The impact of TLR2 and TLR4 signals to fight against *Cryptococcus gattii* infection is unknown, which is a fungal pathogen that preferentially infects the lung of immunocompetent individuals. The macrophages initiate an immune response to combat the *C. gattii*, then we evaluated in RAW 264.7 cell the effect of TLR2 and TLR4 agonists on the macrophage polarization dynamic and the impact on the growth of *C. gattii*.

**Methods and Results:**

We demonstrated that P3C4, LPS, and ArtinM induced an increase in the levels of iNOS transcripts in RAW 264.7 cells, whereas the relative expression of arginase-1, Ym-1, and Fizz1 was significantly increased in the presence of IL-4 alone. The effects of TLR2 and TLR4 agonists on repolarization from the M2 to M1 subset was evaluated, and the first stimulus was composed of IL-4 and, after 24 h of incubation, the cells were submitted to a second stimulus of P3C4, LPS, ArtinM, or Medium. These TLR agonists induced the production of TNF-*α* in polarized RAW 264.7 cells to the M2 subset, moreover the measurement of M1/M2 markers using qRT-PCR demonstrated that a second stimulus with LPS for 24 h induced a significant augmentation of levels of iNOS mRNA. This impact of TLR2 and TLR4 agonists in the activation of the RAW 264.7 macrophage was assayed in the presence of *C. gattii*, the macrophages stimulated with TLR2 and TLR4 agonists for 24 h and co-cultured with *C. gattii*, as a second stimulus, reached high levels of TNF-*α* even after incubation with different concentrations of *C. gattii*. The activation of RAW 264.7 cells induced by TLR2 and TLR4 agonists favored the phagocytosis of *C. gattii* and inhibited the growth of yeast in the early period of infection. However, RAW 264.7 cells incubated with *C. gattii* in the presence of TLR2 and TLR4 agonists did not result a significant difference in the colony forming unit (CFU) assay in the early period of *C. gattii* infection, compared to negative control.

**Conclusion:**

Polarized RAW 264.7 cells to the M1 subset with TLR2 and TLR4 agonists did not inhibit the growth of *C. gattii*, whereas robust immunity was identified that could dysregulate host tolerance to this pathogen.

## Introduction

The multiple functions of macrophages in homeostasis and inflammation demonstrate that these cells play a central role in the modulation of immunity response that may be critical in the success of host defense against different pathogens (Shapouri-Moghaddam et al., 2018; Sica & Mantovani, 2012). The macrophage lineage is characterized by plasticity due to the acquisition of distinct functional phenotypes, and two major subsets are frequently evaluated; classical M1 activation and alternative M2 activation. These phenotypes are influenced by micro-environmental stimuli and signals that they encounter in tissues, which determine the efficacy of polarized macrophages in maintaining homeostasis (Shapouri-Moghaddam et al., 2018; Wang, Liang & Zen, 2014). The main stimuli associated with the induction of M1 macrophages are tumor necrosis factor-alfa (TNF-*α*), interferon-gamma (IFN-*γ*), and lipopolysaccharide (LPS), whereas the presence of interleukin (IL) 4 and IL-10 mediate the M2 macrophages (de Sousa, Da Costa Vasconcelos & Quaresma, 2019). High levels of proinflammatory cytokines accompanied by high production of reactive nitrogen and oxygen intermediates belong to the M1 phenotype and ensure strong microbicidal and tumoricidal activity (Davis et al., 2013; Klar et al., 2018; Zhu et al., 2017). In addition, the M1 subset expresses inducible nitric oxide (NO) synthase (iNOS), which is a primary marker for detecting the response of these cells in specific sites (Davis et al., 2013; Klar et al., 2018; Zhu et al., 2017). In contrast, M2 macrophages are involved in tissue remodeling and also have immunoregulatory functions (Gordon & Martinez, 2010), and the cell expression markers are characterized by Arg-1, found in inflammatory zone 1 (Fizz1), chitinase-like molecule (Ym1), and CD206 (Murray & Wynn, 2011; Rath et al., 2014). The unbalance of the M1/M2 phenotype and number and distribution of macrophages contribute to tissue damage and pathology during infections (Bashir et al., 2016). Toll-like receptors (TLR) ligands, such as LPS (TLR4 agonist), palmitoyl-3-cysteine-serine-lysine-4 (Pam3CSK4; TLR2 agonist), and ArtinM (mannose-binding lectin; TLR2/CD14 agonist) are greater inductors of M1 macrophages (daSilva et al., 2017), and these TLR agonists can favor a persisting pro-inflammatory response that contributes to the alteration of host tolerance (Butcher et al., 2018). This capacity of TLR signaling is supported by the finding that M2 macrophages can be re-polarized into macrophages with M1 phenotype following exposure to TLR ligands (Mylonas et al., 2009; Stout et al., 2005).

The role of TLR signaling is already reported to control several fungal infections (Netea et al., 2006); and previous study indicate that TLR4 and TLR9 signals can be involved in the pro-inflammatory response induced by *Cryptococcus gattii* (Schoffelen et al., 2013). *C. gattii*, a causative agent of cryptococcosis, is a fungal pathogen that preferentially infects the pulmonary tissue of immunocompetent individuals, and lung-resident macrophages initiate an immune response to combat the *C. gattii* yeast or desiccated basidiospores that reach the tissue (Ngamskulrungroj et al., 2012). However, the modulation of NO production by macrophages occurs via a major capsular component in *C. gattii* called glucuronoxylomannan (GXM), that can be recognized by TLR2 (Fonseca et al., 2010). In addition, the involvement of TLR2 and TLR4 for host defense against cryptococcosis has been studied in relation to *Cryptococcus neoformans* infection, for which there is no consensus regarding the contributions of TLR2 and TLR4 to immunity response during the establishment of *C. neoformans* infection (Biondo et al., 2005; Nakamura et al., 2006; Yauch et al., 2004). On the other hand, a previous study demonstrated that macrophage polarization has plasticity to match the changes in the cytokine environment, and the maintenance of M1 macrophages upon IFN-*γ* stimulus favored the growth inhibition of *C. neoformans* (Davis et al., 2013). Therefore, the present work evaluated in murine macrophage cell line RAW 264.7 the effects of TLR2 and TLR4 agonists on the macrophage polarization dynamic and the impact on the growth of *C. gattii*.

We found that the RAW 264.7 macrophage was polarized to the M1 phenotype in response to Pam3CSk4, LPS, and ArtinM, whereas IL-4 induced the M2 macrophage. RAW 264.7 cells previously incubated with IL-4 had high levels of TNF-*α* after a second stimulus with TLR2 and TLR4 agonists, and the repolarization from M2 to M1 occurred via TLR4 signal. Pam3CSk4 and LPS-stimulated RAW 264.7 cells maintain high levels of TNF-*α* after a second stimulus with IL-4, demonstrating the persistence of the pro-inflammatory response induced by TLR2 and TLR4 agonists. However, RAW 264.7 cells polarized to M1 subset by TLR2 and TLR4 signals did not ensure the growth inhibition of *C. gattii*. Therefore, the prevalence of M1 macrophages polarized to the outcome of *C. gattii* infection should be balanced in therapeutic strategies evaluated.

## Materials & Methods

### RAW 264.7 cell line and *Cryptococcus gattii*

The peritoneal macrophage cell line, RAW 264.7, was routinely grown in RPMI 1640 (GE Healthcare; Chicago, Illinois, USA) supplemented with 10% fetal bovine serum (FBS), 4 mM L-glutamine, and antibiotics and these cells were cultured at 37 °C in a humidified atmosphere containing 5% CO.

*C. gattii* strain R265 (VGII molecular genotype) was recovered on Sabouraud dextrose agar and incubated at 30 °C for 24 h. One loopful from a single colony was inoculated in Sabouraud dextrose broth and grown for 24 h at 30 °C with constant shaking (150 rpm). Yeast was harvested by centrifugation at 2000 *xg* for 10 min at 25 °C, washed in sterile phosphate-buffered saline (PBS), and counted using China ink in a Neubauer chamber. The concentration of the yeast in each *in vitro* infection is described in the figure legend.

### Macrophage polarization/repolarization in response to Pam3CSK4-P3C4, LPS, and ArtinM

Synthetic triacylated lipoprotein (Pam3CSK4-P3C4) was purchased from Invivogen (catalog code: tlrl-pms; San Diego, CA, USA), and LPS was purchased from Sigma (Sigma-Aldrich, St. Louis, MO, USA). ArtinM was purified as described previously (Da Silva et al., 2020) from the saline extract of *Artocarpus heterophyllus* (jackfruit) seeds through affinity chromatography with immobilized carbohydrate columns. The endotoxin removal from ArtinM solution was performed as described previously (Da Silva et al., 2020).

RAW 264.7 cells were distributed in a 12-well microplate at a concentration of 1 × 10^5^ cells/mL. RAW 264.7 cells were incubated with LPS (0.1 µg/mL), P3C4 (0.1 µg/mL), ArtinM (2.5 µg/mL), IL-4 (40 ng/mL), or medium alone (Medium). After 24 h of incubation, the cell culture supernatants were collected to quantify the levels of TNF-*α* using enzyme-linked immunosorbent assay (ELISA) kits (BD Biosciences, San Diego, CA, USA), and the RNA from macrophages was isolated by TRIzol Reagent (Life Technologies, Carlsbad, CA, USA).

The quantification of transcripts of iNOS and arginase-1 by quantitative reverse transcription polymerase chain reaction (qRT-PCR) in M2 macrophages incubated with TLR2 or TLR4 agonists was performed using RAW 264.7 cells (1 × 10^5^ cells/mL) distributed in a 12-well microplate. Initially, macrophages were incubated with IL-4 (40 ng/mL), which is an inductor of the M2 phenotype, or Medium, as the first stimulus. After 24 h, the cells were submitted to a second stimulus composed of LPS (0.1 µg/mL), P3C4 (0.1 µg/mL), IL-4 (40 ng/mL), or Medium for an additional 24 h.

### Determining the levels of TNF-*α* in M2 macrophages after stimulation with TLR2 or TLR4 agonists

RAW 264.7 cells (1 × 10^4^ cells/mL) distributed in a 96-well microplate. The first stimulus was composed of LPS (0.1 µg/mL), P3C4 (0.1 µg/mL), ArtinM (2.5 µg/mL), IL-4 (10, 40, or 80 ng/mL), or Medium. After 24 h of incubation the levels of TNF-*α* were measured, and an additional 24 h of incubation containing a second stimulus was performed as follows: fresh medium; cells previously stimulated with LPS, P3C4, ArtinM, IL-4, or Medium were restimulated with the same stimulus; macrophages polarized to the M2 subset by stimulation with IL-4 at concentrations of 10 ng/mL, 40 ng/mL, and 80 ng/mL received a second stimulus composed of LPS, P3C4, ArtinM, IL-4, or Medium . The cell culture supernatants were obtained to quantify the levels of TNF-*α* by ELISA.

### Quantification of the levels of TNF-*α* in response to the effect of IL-4 in RAW 264.7 cells previously incubated with P3C4, LPS, or ArtinM

RAW 264.7 cells (1 × 10^4^ cells/mL) were distributed in a 96-well microplate. The first stimulus was composed of LPS (0.1 µg/mL), P3C4 (0.1 µg/mL), ArtinM (2.5 µg/mL), IL-4 (40 ng/mL), or Medium. After 24 h of incubation the levels of TNF-*α* were measured. An additional 24 h of incubation containing a second stimulus was performed as follows: fresh medium; cells previously stimulated with LPS, P3C4, ArtinM, IL-4, or Medium were restimulated with the same stimulus; macrophages incubated with LPS, P3C4, ArtinM, IL-4, or Medium received a second stimulus composed of IL-4 at concentrations of 10 ng/mL, 40 ng/mL, and 80 ng/mL. The levels of TNF-*α* were measured in cell culture supernatants.

### Co-culture of *C. gattii* and RAW 264.7 cells stimulated with P3C4, LPS, or ArtinM

The RAW 264.7 cell line (2 × 10^4^ cells/mL) was plated in a 48-well microplate and rested for 4 h. These cells were incubated with LPS (0.1 µg/mL), P3C4 (0.1 µg/mL), ArtinM (2.5 µg/mL), IL-4 (40 ng/mL), or Medium for 24 h. These cells were co-cultured with *C. gattii* at a concentration of 2 × 10^4^ yeast/mL, or Medium as a negative control, and these conditions were considered a second stimulus. After 24 h of incubation, the levels of TNF-*α* were measured in the cell culture supernatant by ELISA.

In another assay, RAW 264.7 cells (2 × 10^4^ cells/mL) were co-cultured with live or heat-killed yeast of *C. gattii* at a concentration of 2 × 10^3^ yeast/mL in a 48-well microplate at 37 °C in a humidified atmosphere containing 5% CO_2_. Concomitantly, macrophages received LPS (0.1 µg/mL), P3C4 (0.1 µg/mL), ArtinM (2.5 µg/mL), IL-4 (40 ng/mL), or Medium. The cell culture supernatant was collected after 24 h of incubation and the levels of TNF-*α* were quantified by ELISA.

The measurement of relative expression of iNOS and arginase-1 was performed in RAW 264.7 cells (1 × 10^5^ cells/mL) stimulated with LPS (0.1 µg/mL), P3C4 (0.1 µg/mL), ArtinM (2.5 µg/mL), IL-4 (40 ng/mL), or Medium, and concomitantly co-cultured with live yeast of *C. gattii* (1 × 10^5^ yeast/mL). After 24 h of incubation at 37 °C in a humidified atmosphere containing 5% CO_2_, the RNA from macrophages was isolated by TRIzol Reagent (Life Technologies, Carlsbad, CA, USA). The quantification of transcripts of iNOS and arginase-1 by qRT-PCR was performed as described below in the quantitative reverse transcription topic.

### Fungistatic effect of RAW 264.7 cells stimulated with P3C4, LPS, or ArtinM on *C. gattii*

The murine RAW 264.7 cell line at a concentration of 1 × 10^5^ cells/mL was distributed in a 24-well microplate and rested for 4 h at 37 °C in a humidified atmosphere containing 5% CO_2_. Macrophages were stimulated with LPS (0.1 µg/mL), P3C4 (0.1 µg/mL), ArtinM (2.5 µg/mL), IL-4 (40 ng/mL), or Medium for 24 h. Then, 2 × 10^4^ yeast/mL of *C. gattii* was added to the culture of previously stimulated macrophages, and the co-culture was incubated for 24 h at 37 °C in a humidified atmosphere containing 5% CO_2_. *C. gattii* yeasts were plated in the absence of macrophages, which were considered the control of growth of *C. gattii* under the same conditions. The monolayer culture was detached and macrophages were lysed using sterile water and the suspension was mixed with supernatant to quantify the growth of *C. gattii* by the colony forming unit (CFU) assay that was expressed as CFU/mL

### Phagocytosis and antifungal activity of RAW 264.7 cells stimulated with P3C4, LPS, or ArtinM and co-cultured with *C. gattii*

RAW 264.7 cells at a concentration of 1 × 10^4^ cells/mL was distributed in a 24-well microplate and rested for 4 h at 37 °C in a humidified atmosphere containing 5% CO_2_. Macrophages were stimulated with LPS (0.1 µg/mL), P3C4 (0.1 µg/mL), ArtinM (2.5 µg/mL), IL-4 (40 ng/mL), or Medium. After 24 h, live yeast of *C. gattii* (1 × 10^5^ yeast/mL) was added to the culture and the co-culture was incubated for 5 h at 37 °C. Then, the samples were washed twice with PBS to remove nonadherent yeast and the cells were lysed with sterile water. This suspension was plated onto Sabouraud agar plate for CFU quantification. For antifungal activity assay, once the samples were washed twice with PBS to remove nonadherent yeast a fresh medium was added, and incubation was continued for an addition 5 h. After incubation, cells were lysed with sterile water and the fungal cells were plated onto Sabouraud agar plate for CFU quantification.

### Quantitative reverse transcription PCR and cytokine measurement

RNA from macrophages stimulated for 24 h was isolated using the TRIzol Reagent (Life Technologies, Carlsbad, CA, USA), according to the manufacturer’s protocol. The total RNA was reverse-transcribed into complementary DNA (cDNA) using an iScript™ cDNA Synthesis Kit (Bio-Rad). Real-time PCR was performed in 10 µL reactions using 2x qPCRBIO SyGreen Mix Separate-ROX (PCRBiosystems; Wayne, PA, USA), cDNA (10-25 ng), and 0.3 µM of the primer mix. All reactions were performed on a Bio-Rad CFX96 Real-Time Detection System (Bio-Rad) under the following conditions: 95 °C for 2 min, and 40 cycles of 95 °C for 5 s/60 °C for 30 s. Gene expression was quantified using the Δ ΔCt method and normalized to *β*-actin expression. The PCR primers used were: *β*-actin (F-CCTAAGGCCAACCGTGAAAA, R-GAGGCATACAGGGACAGCACA), Ym-1 (F-TCACAGGTCTGGCA ATTCTTCTG, R-ACTCCCTTCTATTGGCCTGTCC), Arginase-1 (F-GTTCCCAGATGTACCAGGATTC, R-CGATGTCTTTGGCAGATATGC), Fizz1 (F-CCTGAGATTCTGCCCCAGGAT, R-TTCACTGGGA CCATCAGCTGG), and iNOS2 (F-CCGAAGCAAACATCACATTCA, R-GGTCTAAAGGCTCCGGGCT).

The supernatants were used for the quantification of TNF-a levels by ELISA according to the manufacturer’s protocol using Ab pairs purchased from BD Biosciences (Pharmingen, San Diego, CA, USA). Concentrations were determined relative to standard curves prepared from recombinant murine cytokines. Absorbances were read at 450 nm in a PowerWave X microplate scanning spectrophotometer (BioTek Instruments, Inc.).

### Statistical analysis

Results are presented as means ± SD. All data were analyzed using GraphPad Prism v.7.0 (GraphPad Software, San Diego, CA, USA). The normality test of all statistical determinations were analyzed by the Shapiro–Wilk normality test. For datasets with a non-normal distribution, the Kruskal-Wallis test was used for experiments with three or more groups. Differences between the means of groups were evaluated by one-way ANOVA followed by Tukey’s multiple comparisons test or the Kruskal–Wallis test followed by Dunn’s multiple comparisons test. Differences at *p* < 0.05 were considered statistically significant.

## Results

### Role of TLR2 and TLR4 agonists in the polarization and repolarization of RAW 264.7 cells

**Figure 1:**
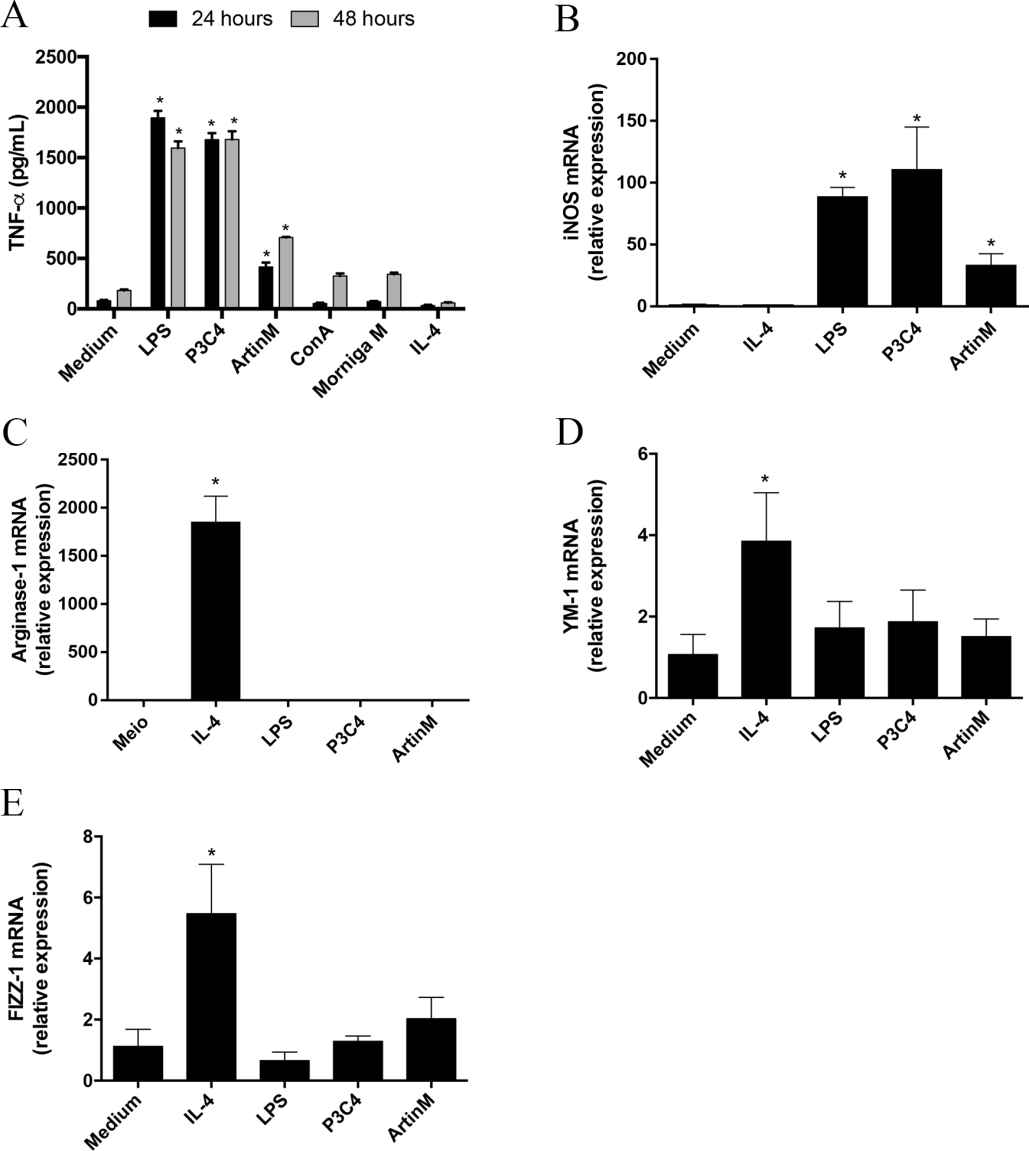
RAW 264.7 cells stimulated with TLR2 and TLR4 agonists differentiated to M1 subset. RAW 264.7 cells at a concentration of 1 ×10^5^ cells/mL were incubated with LPS (0.1 mg/mL), P3C4 (0.1 µg/mL), ArtinM (2.5 mg/mL), IL-4 (40 ng/mL), or medium alone (Medium). (A) After 24 h of incubation, the levels of TNF-a were measured in the cell culture supernatants by ELISA. (B–E) The RNA samples extracted from these cells were reverse-transcribed into cDNA and analyzed for the expression of iNOS2, Arginase-1, Ym-1, and Fizz-1 by qRT-PCR. The levels of transcripts and TNF-*α* in RAW 264.7 cells stimulated with LPS, P3C4, ArtinM, and IL-4 were compared with those of cells in Medium. Data are shown as means ±  SD, and ^∗^*p* < 0.05, and the difference between groups was evaluated by one-way ANOVA followed by Tukey’s multiple comparisons test. Gene expression was quantified by using the Δ ΔCt method and normalized to *β*-actin expression.

Macrophage polarization occurs in different sources of macrophages and the microenvironment and innate immune receptors are major inductors in differentiation to M1 or M2 subsets (Mantovani et al., 2004; Wang, Liang & Zen, 2014). The modulation of M1- and M2-type macrophage polarization can be orchestrated by *C. gattii* compounds through interactions with receptors on the cell surface (Ueno et al., 2019). In this context, carbohydrates located in the *C. gattii* capsule can inhibit TLR2 and TLR4 signaling, which is a mechanism to subvert the host immune response. Then, we evaluated the effect of TLR2 and TLR4 agonists on the maintenance of polarized RAW 264.7 cells into the M1 subset over time during *C. gattii* infection. Initially, we demonstrated that P3C4, LPS, and ArtinM induced a significant production of TNF- *α* by RAW 264.7 cells after 24 and 48 h of incubation (Fig. 1A). The impact of TLR2 and TLR4 agonists in M1/M2 polarization was investigated by measuring the mRNA levels of M1/M2 markers, and RAW 264.7 cells stimulated with TLR2 and TLR4 agonists had a significant increase in the levels of iNOS transcripts (Fig. 1B), whereas the relative expression of arginase-1, Ym-1, and Fizz-1 was significantly altered in the presence of IL-4 alone (Figs. 1C–1E).

**Figure 2:**
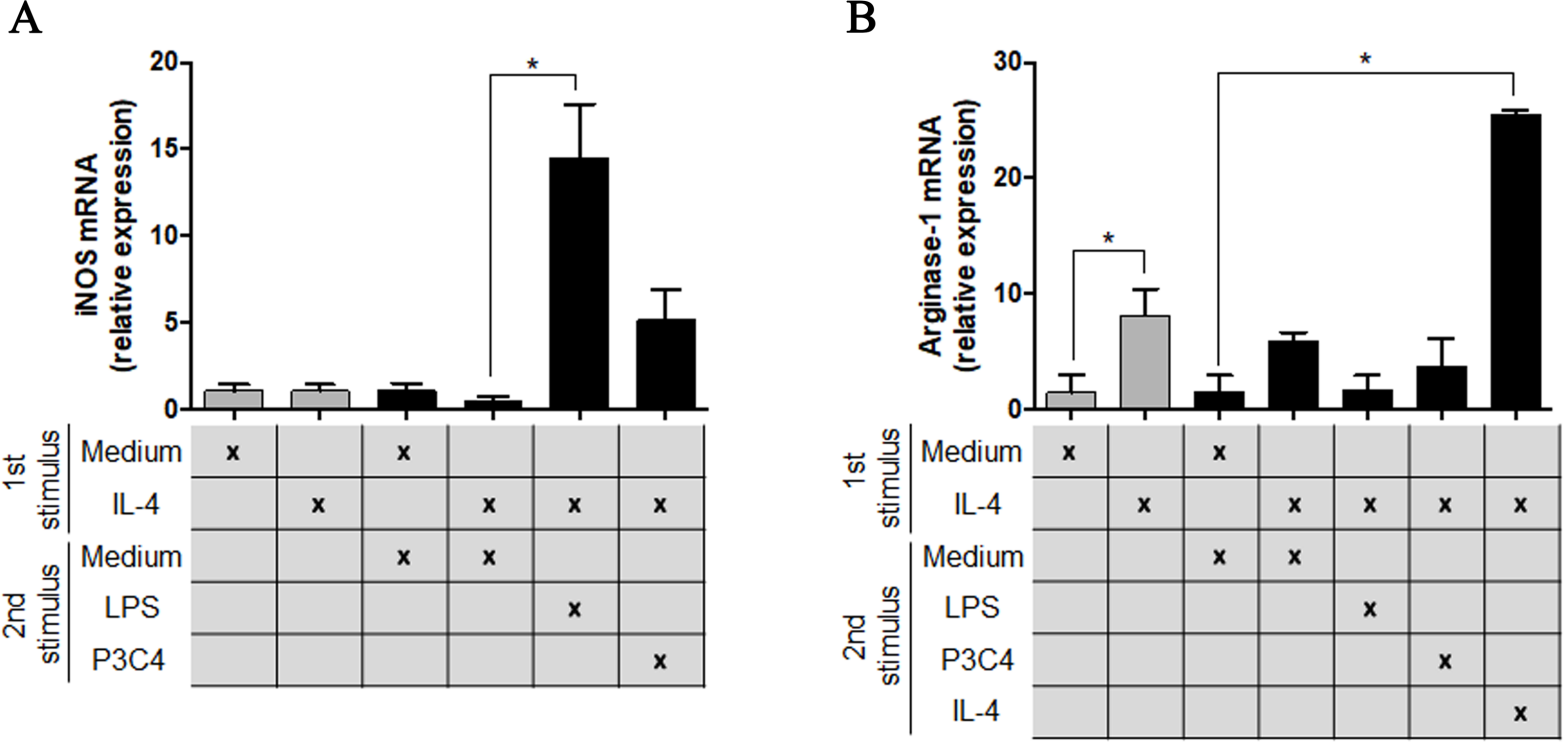
The M2 subset of RAW 264.7 cells was repolarized to the M1 phenotype in the presence of LPS stimulus. RAW 264.7 cells (1 ×10^5^ cells/mL) were incubated with IL-4 (40 ng/mL) or medium alone (Medium), and the relative expression of iNOS (A) and arginase-1 (B) was measured by RT-PCR after 24 h of incubation (gray bar). A second stimulus composed of LPS (0.1 mg/mL), P3C4 (0.1 µg/mL), IL-4 (40 ng/mL), or Medium was performed, and the transcripts for iNOS (A) and arginase-1 (B) were quantified after 24 h of incubation. The values are expressed as means ±  SD and ^∗^*p* < 0.05, according to the Kruskal–Wallis test followed by Dunn’s multiple comparisons test.

To understand the potential of TLR2 and TLR4 agonists in the modulation of M2 macrophages, the RAW 264.7 cell line was incubated with different concentrations of IL-4 and, after 24 h of incubation, the cells were stimulated with P3C4, LPS, or ArtinM. This hypothesis was investigated via the measurement of M1/M2 markers in polarized RAW 264.7 cells to the M2 phenotype that received a second stimulus with TLR2 and TLR4 agonists for 24 h. The transcripts of iNOS and arginase-1 were quantified by qRT-PCR and only LPS induced a significant augmentation of levels of iNOS mRNA in macrophages (Fig. 2A), whereas the levels of arginase-1 mRNA in macrophages stimulated with P3C4 or LPS had been lower than those incubated with Medium (Fig. 2B). These findings demonstrate that TLR2 and TLR4 agonists can act in macrophage repolarization from the M2 to M1 phenotype.

### TNF-*α* production induced by TLR2 and TLR4 agonists in RAW 264.7 cells is not affected by live yeast of *C. gattii*

**Figure 3:**
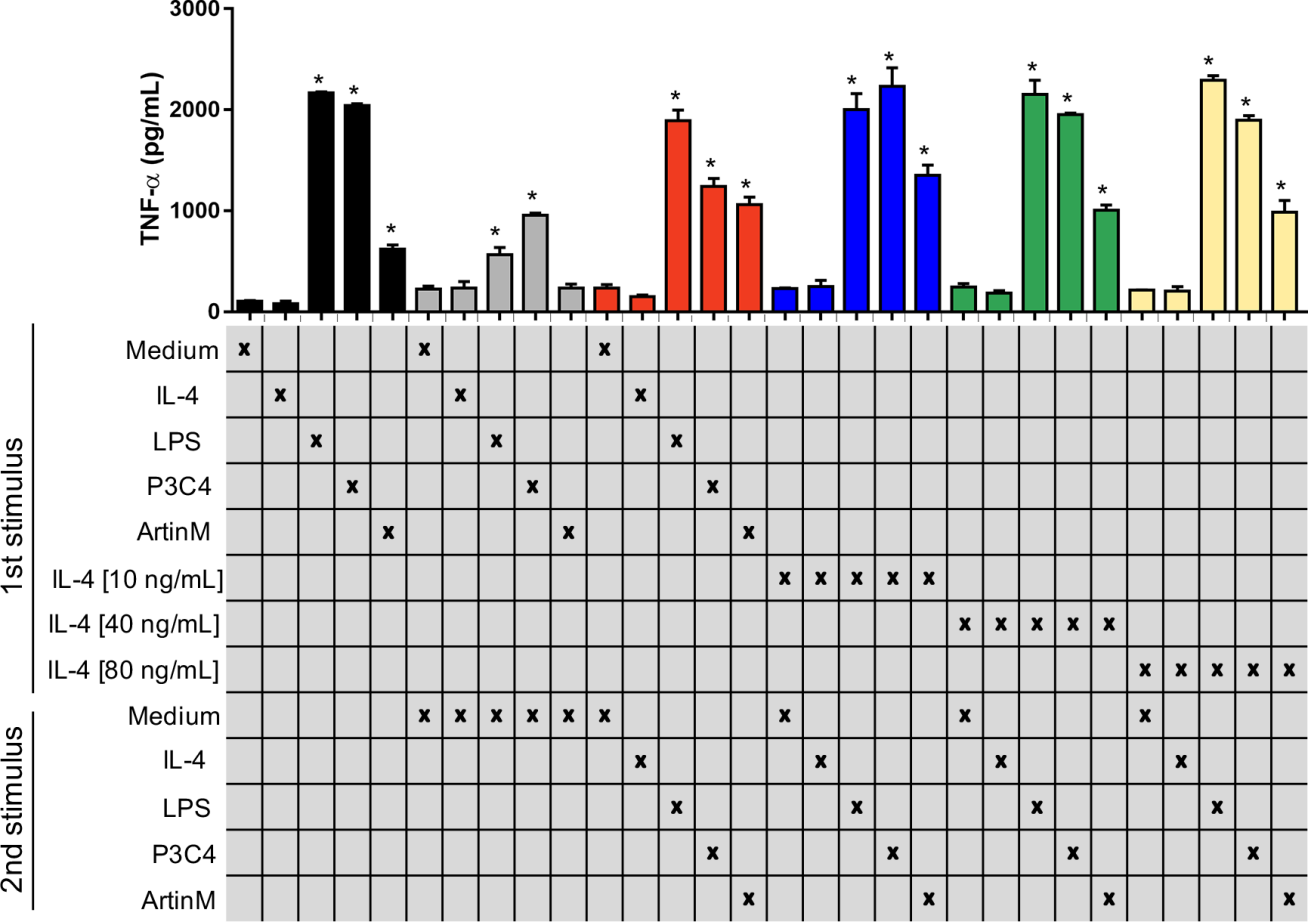
RAW 264.7 macrophages previously incubated with IL-4 began to produce TNF-a in the presence of TLR2 and TLR4 agonists. RAW 264.7 cells (1 ×10^4^ cells/mL) received the first stimulus composed of LPS (0.1 µg/mL), P3C4 (0.1 mg/mL), ArtinM (2.5 µg/mL), IL-4 (10, 40, or 80 ng/mL), or medium alone (Medium). After 24 h of incubation, the levels of TNF-a were measured in the cell culture supernatants (black bar). An additional 24 h of incubation was done using as a second stimulus as follows: fresh medium (gray bar); (red bar) restimulated with LPS (0.1 µg/mL), P3C4 (0.1 mg/mL), ArtinM (2.5 µg/mL), IL-4 (40 ng/mL), or Medium; (blue bar) IL-4 at a concentration of 10 ng/mL; (green bar) IL-4 at a concentration of 40 ng/mL; (yellow bar) IL-4 at a concentration of 80 ng/mL. The levels of TNF-*α* were measured in the cell culture supernatants by ELISA. Results are shown as means ±  SD, and ^∗^*p* < 0.05, according to the Kruskal-Wallis test followed by Dunn’s multiple comparisons test. * Compared to the Medium.

Previous studies have reported that *C. gattii* modulates the host immune response using capsule compounds and intracellular proteins released (Fonseca et al., 2010), and innate immune receptors are potential targets in the suppression of the immune response induced by *C. gattii*. The impact of TLR2 and TLR4 agonists in the activation of the RAW 264.7 cells was assayed in the presence of an inflammatory regulator, such as IL-4, which was evaluated using two different approaches as follows: (i) cells were incubated with IL-4 at different concentrations (10-80 ng/mL) and were submitted to a second stimulus of P3C4, LPS, or ArtinM (Fig. 3); (ii) cells were stimulated with LPS, P3C4, or ArtinM for 24 h, and a second stimulus with IL-4 (10 to 80 ng/mL) was performed (Fig. S1). In both approaches the production of TNF-*α* was measured, and we observed that macrophages previously incubated with IL-4 at different concentrations (10–80 ng/mL) showed high levels of TNF-*α* after 24 h of incubation with P3C4, LPS, or ArtinM as second stimulus, compared to Medium (Fig. 3, blue, green, and yellow bars). In addition, there was no significant difference in the levels of TNF-*α* induced by TLR2 and TLR4 agonists between macrophages previously incubated with IL-4 at different concentrations (Fig. 3, blue, green, and yellow bars). In the other hand, the first stimulus with LPS, P3C4, or ArtinM induced high levels of TNF-*α* in the RAW 264.7 cell after 24 h of incubation (Fig. S1, black bar), and these levels were reduced after a second stimulus with IL-4 in a dose-dependent manner (Fig. S1, blue, green, and yellow bars). Taken together, these findings suggest that the TLR2 and TLR4 agonists-activated RAW 264.7 cell induce a proinflammatory response even in the presence of inflammatory regulator.

**Figure 4:**
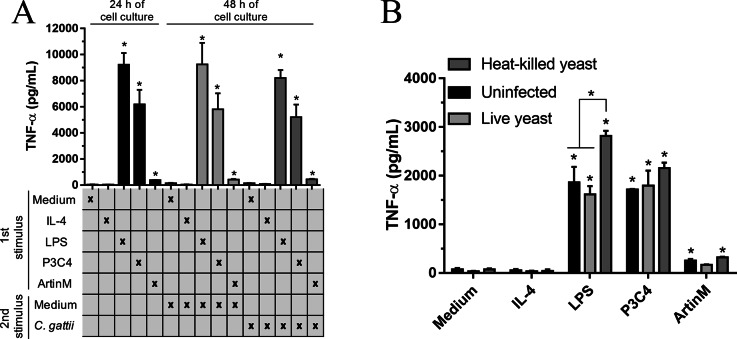
TLR2 and TLR4 agonists induced the production of TNF-*α* in RAW 264.7 cells co-cultured with *C. gattii*. (A) The RAW 264.7 cell line (2 ×10^4^ cells/mL) was plated in a 48-well microplate and incubated with LPS (0.1 mg/mL), P3C4 (0.1 µg/mL), ArtinM (2.5 mg/mL), IL-4 (40 ng/mL), or medium alone (Medium). After 24 h, RAW 264.7 cells were co-cultured with *C. gattii* (MOI 1:1) or Medium as a negative control for an additional 24 h of incubation. (B) RAW 264.7 cells (2 ×10^4^ cells/mL) were co-cultured with live or heat-killed yeast of *C. gattii* at a concentration of 2 ×10^3^ yeast/mL in a 48-well microplate for 24 h of incubation. LPS (0.1 µg/mL), P3C4 (0.1 mg/mL), ArtinM (2.5 µg/mL), IL-4 (40 ng/mL), or Medium were added concomitantly to the co-cultured with C. gattii. (A and B) The levels of TNF-a were performed in the cell culture supernatant by ELISA, and results are shown as means ±  SD ^∗^*p* < 0.05, according to the Kruskal–Wallis test followed by Dunn’s multiple comparisons test. * Compared to the Medium.

The immunomodulation induced by TLR agonists in RAW 264.7 cells was also evaluated after co-culture with *C. gattii* yeasts. The macrophages were stimulated with TLR2 and TLR4 agonists in the absence of *C. gattii* for 24 h and an additional 24 h of incubation in the presence of *C. gattii*. The levels of TNF-*α* were measured in the culture supernatant, and the cells incubated with TLR2 and TLR4 agonists for 24 h and co-cultured with *C. gattii* as a second stimulus induced high levels of TNF-*α* in response to stimulation with LPS and P3C4, compared to the Medium (Fig. 4A). In other assay, the effect of *C. gattii* on the levels of TNF-*α* produced was also tested in the RAW 264.7 cells concomitantly stimulated with TLR2 or TLR4 agonists and co-cultured with *C. gattii* (live or heat-killed yeast), and after 24 h the levels of TNF-*α* were measured. The LPS and P3C4-activated RAW 264.7 cells reached high levels of TNF- *α* even in the presence of *C. gattii*, and the live yeast of *C. gattii* did not decrease the production of TNF-*α* induced by RAW 264.7 cells stimulated with TLR2 and TLR4 agonists (Fig. 4B). However, the levels of TNF-*α* induced by LPS stimulus had a significant increase in the presence of heat-killed yeast of *C. gattii*, compared to RAW 264.7 cells co-cultured with or without live yeast of *C. gattii* (Fig. 4B). Therefore, the improvement in the production of TNF-*α* by RAW 264.7 cells in response to LPS stimulus occurred by heat-killed yeast of *C. gattii*.

### RAW 264.7 cells stimulated with TLR2 and TLR4 agonists had not a fungistatic effect on *C. gattii* infection due the alteration in the balance of iNOS/Arginase-1 expression

**Figure 5:**
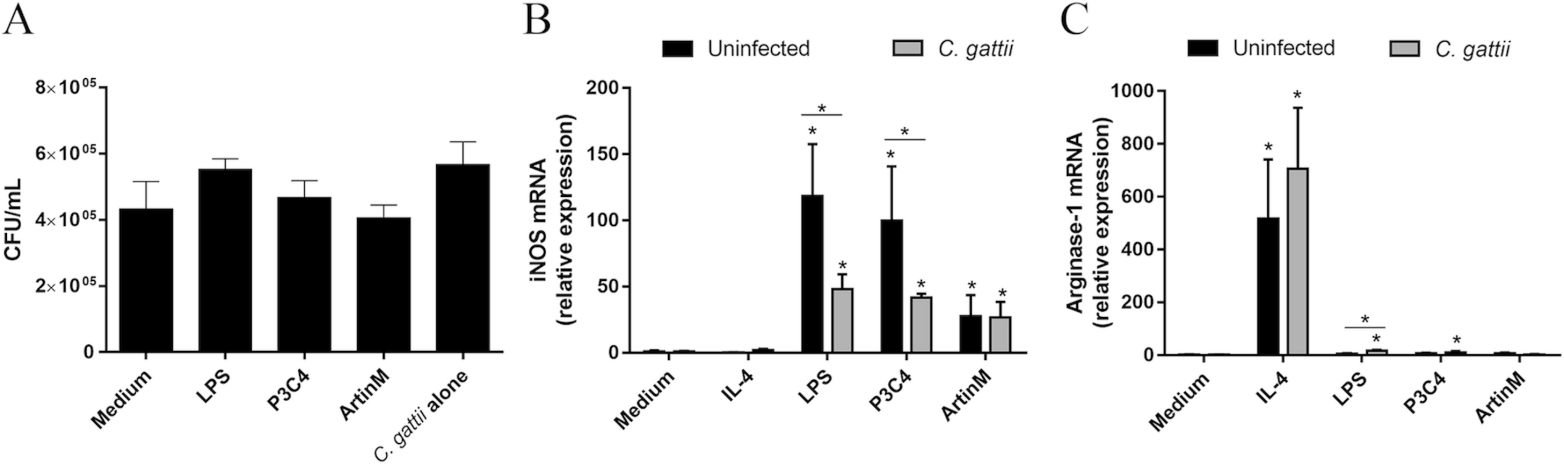
*C. gattii* growth was not reduced by RAW 264.7 cells incubated with TLR2 and TLR4 agonists due to unbalance of iNOS/Arg-1 expression. (A) 2 ×10^4^ yeast/mL of *C. gattii* was added to a culture of RAW 264.7 cells (1 ×10^5^ cells/mL) previously stimulated with LPS (0.1 mg/mL), P3C4 (0.1 µg/mL), ArtinM (2.5 mg/mL), IL-4 (40 ng/mL), or medium alone (Medium) for 24 h. As a control of the growth of *C. gattii*, the yeasts were incubated in the absence of macrophages (*C. gattii* alone). The colony forming unit (CFU) assay was performed using a monolayer culture detached and mixed with supernatant, and the results were expressed as CFU/mL. (B–C) Measurement of relative expression of iNOS and arginase-1 in RAW 264.7 cells (1 ×10^5^ cells/mL) stimulated with LPS (0.1 mg/mL), P3C4 (0.1 µg/mL), ArtinM (2.5 mg/mL), IL-4 (40 ng/mL), or Medium. Moreover, the cells were concomitantly co-cultured with live yeast of *C. gattii* (1 ×10^5^ yeast/mL). After 24 h of incubation, the RNA from macrophages was extracted for quantification of transcripts of iNOS and arginase-1 by qRT-PCR. (A, B, and C) The values are expressed in means ±  SD, and ^∗^*p* < 0.05, according to the Kruskal–Wallis test followed by Dunn’s multiple comparisons test.

Proinflammatory mediators are required in several intracellular pathogen infections and previous studies have reported the importance of proinflammatory immune responses to fight cryptococcosis (Kumaresan, daSilva & Kontoyiannis, 2017). As LPS and P3C4 modulate RAW 264.7 cells to the M1 phenotype, this study evaluated the co-culture of RAW 264.7 cells with *C. gattii* in the presence of TLR2 and TLR4 agonists. The macrophages were incubated with LPS, P3C4, ArtinM, or Medium for 24 h, and infection with *C. gattii* yeasts (1:100 *C. gattii*/macrophage ratio) was performed. After 24 h of co-culture, the growth of *C. gattii* was quantified by CFUs and the results provided by co-incubation of unstimulated RAW 264.7 cells (Medium) with *C. gattii* did not significantly differ from those obtained after stimulation with LPS, P3C4, or ArtinM. The *C. gattii* culture alone in the absence of RAW 264.7 cells (*C. gattii* alone) was performed as control, and the growth of *C. gattii* yeasts in unstimulated RAW 264.7 cells (Medium) did not differ from those obtained from *C. gattii* alone (Fig. 5A). The outcome of *C. gattii* infection was not strongly associated with M1 macrophages polarized by TLR2 and TLR4 agonists.

To understand the effect of *C. gattii* infection in the polarization of RAW 264.7 cells induced by TLR2 and TLR4 agonists, these cells were stimulated with IL-4, LPS, P3C4, and ArtinM concomitantly or not (uninfected) with live yeast of *C. gattii*. After 24 h, the mRNA levels of iNOS and arginase-1 were measured by RT-PCR. *C. gattii*-infected RAW 264.7 cells after stimulation with TLR2 and TLR4 agonists had a significant decrease in the levels of iNOS transcripts compared to uninfected macrophages (Fig. 5B), whereas the relative expression of arginase-1 was significantly augmented in RAW 264.7 cells infected with *C. gattii* and stimulated with LPS (Fig. 5C). These findings demonstrated that the *C. gattii* infection is able to regulate the iNOS expression induced by TLR2 and TLR4 agonists, which can influence the microbicidal activity of macrophage favoring the growth of yeast.

### RAW 264.7 cells stimulated with TLR2 and TLR4 agonists inhibit the growth of *C. gattii* in the early period of infection

**Figure 6:**
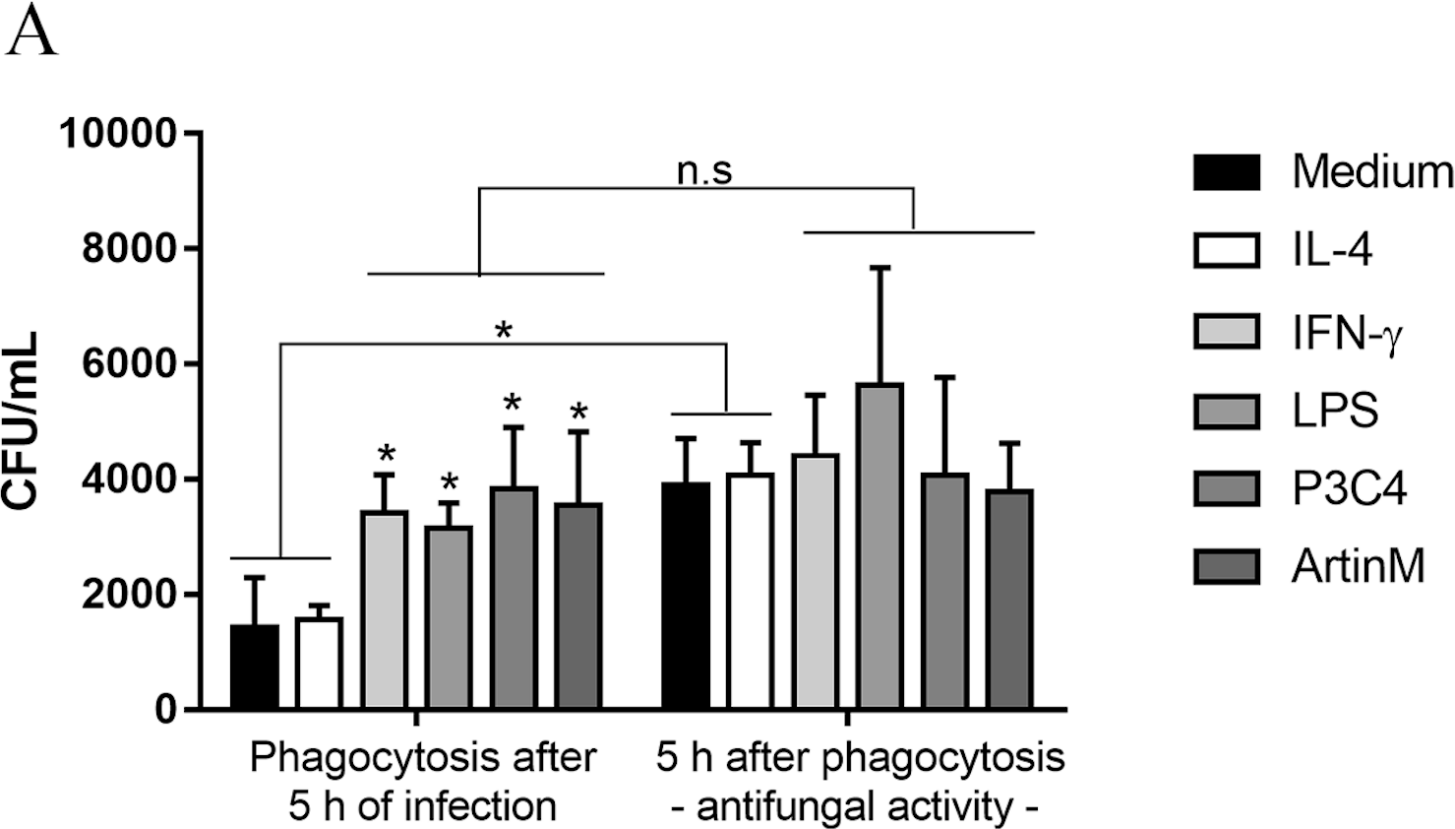
RAW 264.7 macrophages stimulated with TLR2 and TLR4 agonists had improved the phagocytosis and antifungal activity in early period of *C. gattii* infection. RAW 264.7 macrophages (1 ×10^4^ cells/mL) were stimulated with LPS (0.1 µg/mL), P3C4 (0.1 mg/mL), ArtinM (2.5 µg/mL), IL-4 (40 ng/mL), or Medium for 24 h. Live yeast of *C. gattii* (1 ×10^5^ yeast/mL) was co-cultured with macrophages for 5 h at 37 °C. For phagocytosis assay, the nonadherent yeast were removed after washed twice with PBS, and the macrophages lysed were plated onto Sabouraud agar plate for CFU quantification. For antifungal activity, fresh medium was added once nonadherent yeast was removed and an addition 5 h was continued to measure the CFU. The values are expressed in means ±  SD, and ^∗^*p* < 0.05, according to the Kruskal-Wallis test followed by Dunn’s multiple comparisons test.

This work also investigated the impact of TLR2 and TLR4 agonists in the phagocytic activity of RAW 264.7 cells in the presence of *C. gattii*. The cells were incubated with IL-4, IFN- *γ*, LPS, P3C4, or ArtinM for 24 h previously the addition of *C. gattii*, and after 5 h of *in vitro* infection the non-adherent yeasts were removed. The level of phagocytosis was performed by CFU and the activation of RAW 264.7 cells induced by TLR2 and TLR4 agonists and IFN-*γ* improved the phagocytosis of *C. gattii*, compared to unstimulated macrophages (Medium) (Fig. 6). Then, the capacity of RAW 264.7 cells to control the growth of *C. gattii* in response to stimulation with TLR2 and TLR4 agonists was performed. To evaluated the antifungal activity of macrophages previously stimulated the cells were submitted to conditions described above for phagocytosis assay. Once the non-adherent yeasts were removed, fresh medium was added and incubation was continued for an additional 5 h and macrophage lysate was plated for CFU determination. The quantification of *C. gattii* in RAW 264.7 cells previously incubated with medium or IL-4 had a significant increase compared to CFU quantified after phagocytosis (Fig. 6). However, the stimulation of RAW 264.7 cells with IFN-*γ*, LPS, P3C4, or ArtinM did not result a significant difference compared to the adherent yeast measured in the phagocytosis assay (Fig. 6). Then, pro-inflammatory immune response induced in RAW 264.7 cells by TLR2 and TLR4 agonists favors the uptake of *C. gattii* promoting a control of growth of yeast in the early stage of infection.

## Discussion

The activation of macrophages in response to TLR agonists, in which LPS and Pam3CSK4 are considered the major inductors of pro-inflammatory mediators, is widely studied (Schlaepfer et al., 2014). Moreover, the capacity of TLR2 and TLR4 agonists in the induction of the M1 phenotype has been investigated in macrophages from different sources (Feng et al., 2020; Kulsantiwong et al., 2017; Wang et al., 2020). However, the impact of TLR2 and TLR4 agonists on the activation of macrophages previously regulated by anti-inflammatory cytokine or *C. gattii* infection requires further investigation. In this line, the current study adopted the peritoneal macrophage RAW 264.7 cell line to demonstrate that TLR2 (P3C4 and ArtinM) and TLR 4 (LPS) agonists are great inductors of M1 macrophages, and P3C4 and LPS are able to support the production of TNF-*α* by macrophages upon the presence of immunosuppressor agent. Furthermore, LPS induced repolarization from the M2 to M1 subset. The effect of TLR2 and TLR4 agonists in RAW 264.7 cells ensures the production of high levels of TNF-*α* in the co-culture with *C. gattii*. This immunomodulatory activity promoted by TLR2 and TLR4 agonists in macrophages did not control the *in vitro C. gattii* growth. With the modulation of macrophages to the pro-inflammatory immune response by LPS and P3C4 over time, *C. gattii* infection did not improve host resistance and could be a trigger to alter host tolerance.

TLR2 and TLR4 are relevant in the detection of fungal components, such as zymosan, O-linked mannans, and fungal DNA (Taghavi et al., 2017). Previous studies have investigated the role of TLR2 and TLR4 signaling in *C. neoformans* infection, and the absence of TLR2 and TLR4 has been found to compromise the host response (Biondo et al., 2005; Yauch et al., 2004). Additionally, previous studies have demonstrated that TLR2 and/or TLR4 signals play a limited role in host defense against *C. neoformans* (Biondo et al., 2005; Nakamura et al., 2006). The role of TLR2 and TLR4 in the control of *C. gattii* infection have not yet been evaluated, and previous study demonstrated that the blocking TLR4 significantly decreased the production of IL-1 *β* in response to *C. gattii* stimulation (Schoffelen et al., 2013). Then, TLR2 and TLR4 agonists can be considered in the induction of pro-inflammatory response possibly affected by C. gattii infection. In this context, Fonseca et al. (2010) demonstrated that GXM polysaccharide from serotype B *C. gattii* efficiently induced the TLR2-mediated response (Fonseca et al., 2010). These authors also found that GXM fractions from *C. gattii* serotype B strains can elicit NO production by macrophages, suggesting that soluble GXM can favor M1 polarization via TLR2 signaling. Taken together, TLR2 and TLR4 signals, independently of their limited role in host response against cryptococcosis, are useful in the modulation of innate immune response over time during *Cryptococcus* spp. infection. We selected the mouse macrophage cell line RAW 264.7 to investigate the role of Pam3CSk4, ArtinM (TLR2 agonists), and LPS (TLR4 agonist) in macrophage repolarization for the host response against *C. gattii* infection. This study evidenced the prevalence of M1 markers in RAW 264.7 cells upon TLR2 and TLR4 stimuli, and previous studies have reported the M1 polarization in macrophages from distinct sources in response to TLR2 and TLR4 agonists (Mantovani et al., 2004; Montoya et al., 2019; Sica & Mantovani, 2012). The development of the M1 phenotype is critical for controlling *C. neoformans* infection due to the oxidative burst produced by M1 macrophages, whereas the M2 subset is not antimicrobial against *C. neoformans* (Arora et al., 2011; Johnston & May, 2013). In this context, we demonstrated that TLR2 and TLR4 agonists induced high levels of TNF-*α* in RAW 264.7 cells previously polarized to the M2 phenotype; moreover, LPS induced macrophage repolarization from the M2 to M1 phenotype. These mechanisms triggered by TLR2 and TLR4 agonists are necessary to fight against the *C. neoformans* infection, in which Davis et al. (2013) showed that M2 polarized macrophages can re-polarize to the M1 phenotype, maintaining the functional anti-cryptococcal activity (Davis et al., 2013). However, the mechanisms of host resistance to *C. gattii* infection may not be strictly associated with the M1 macrophages, as there have been no previous studies, to our knowledge, on this topic. We found that the absence of iNOS improves the survival of *C. gattii*-infected mice (Oliveira-Brito et al., 2020), and the high levels of NO induced by *C. gattii* mediated the apoptosis of inflammatory cells, compromising the control of cryptococcosis (Chiapello et al., 2008). Therefore, the strength of TLR2 and TLR4 agonists in M1 macrophage polarization should be evaluated carefully in host resistance within each infection model.

This study used TNF-*α* levels to measure the capacity of TLR2 and TLR4 agonists to modulate the macrophages challenged with distinct stimulus, as TNF-*α* biosynthesis is strictly related to TLR signaling and the production of TNF-*α* is a M1 marker currently being evaluated (Arango Duque & Descoteaux, 2014). The production of TNF-*α* is useful to understand the effects of TLR2 and TLR4 agonists on the maintenance of pro-inflammatory responses or on macrophage repolarization. The pro-inflammatory response and its mechanisms have been widely studied in *C. neoformans* infection, and most studies have used immunomodulator agents polarize toward the M1 profile. However, the success in the balance between pro- and anti-inflammatory responses is an effective way to address the control of cryptococcosis. This hypothesis is supported by the relationship between disease tolerance and resistance, in which the balance between them facilitates efficient pathogen clearance with an acceptable degree of immunopathology (Soares, Teixeira & Moita, 2017). The interaction of fungal virulence and dysregulated host immune response is associated with the pathogenesis of cryptococcal disease, and *C. gattii* is a pathogen that causes disease in hosts with weak or robust immunity (Casadevall & Pirofski, 2003; Casadevall & Pirofski, 2015; Pirofski & Casadevall, 2017). This means that the capacity of TLR2 and TLR4 agonists to induce a pro-inflammatory response and reduce the prevalence of M2 macrophages affects host tolerance to *C. gattii*. In addition, host resistance is not improved with TLR2 and TLR4 agonists against *in vitro C. gattii* infection based on our results, which showed a deficiency in the fungistatic effect on *C. gattii* infection by TLR-stimulated macrophages.

## Conclusions

In conclusion, RAW 264.7 cells stimulated with TLR2 and TLR4 agonists did not experience a fungistatic effect on *C. gattii* infection, whereas robust immunity was identified that could dysregulate host tolerance to this pathogen. This study provides new perspective on therapeutic strategies with a focus on host resistance and tolerance mechanisms in a balanced manner that could have the potential to improve disease outcomes.

##  Supplemental Information

10.7717/peerj.10295/supp-1 Supplemental Information 1 Production of TNF- *α* in RAW 264.7 macrophages stimulated with TLR2 and TLR4 agonists was induced upon IL-4 stimulus RAW 264.7 cells (1 ×10^4^ cells/mL) received a first stimulus containing LPS (0.1 µg/mL), P3C4 (0.1 mg/mL), ArtinM (2.5 µg/mL), IL-4 (40 ng/mL), or medium alone (Medium). After 24 h of incubation, the cell culture supernatants were collected to measure the levels of TNF- *α* by ELISA (black bar). An additional 24 h of incubation a second stimulus was given as follows: (gray bar) fresh medium; (red bar) incubated with LPS (0.1 µg/mL), P3C4 (0.1 mg/mL), ArtinM (2.5 µg/mL), IL-4 (40 ng/mL), or Medium; (blue bar) IL-4 at a concentration of 10 ng/mL; (green bar) IL-4 at a concentration of 40 ng/mL; (yellow bar) IL-4 at a concentration of 80 ng/mL. After 24 h of culture, the quantification of levels of TNF- *α* was performed in cell culture supernatants by ELISA. The values are expressed in means ±  SD and ^∗^*p* < 0.05, according to the Kruskal–Wallis test followed by Dunn’s multiple comparisons test. * Compared to the Medium.

10.7717/peerj.10295/supp-2 Supplemental Information 2 Raw Data

## Additional Information and Declarations

### Data Availability

The following information was supplied regarding data availability: The raw measurements are available in the Supplementary File.
