# Supplementary figures and images for: Pro-inflammatory response ensured by LPS and Pam3CSK4 in RAW 264.7 cells did not improve a fungistatic effect on Cryptococcus gattii infection
Source: PeerJ. 2020 Nov 25;8:e10295. doi: 10.7717/peerj.10295 (PMC7698691; doi:10.7717/peerj.10295)

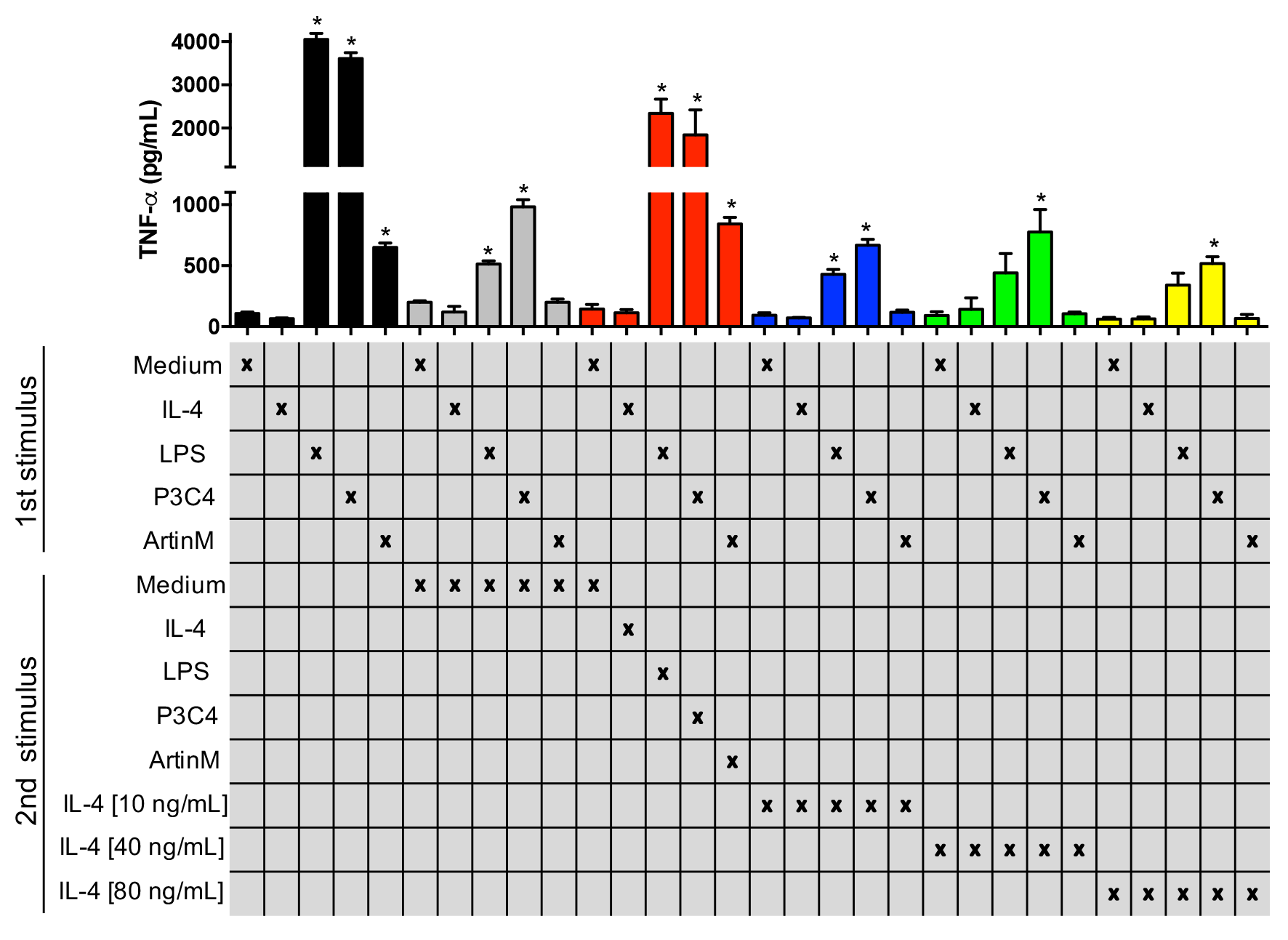

Supplement: Supplemental Information 1 — RAW 264.7 cells (1 ×10^4^ cells/mL) received a first stimulus containing LPS (0.1 µg/mL), P3C4 (0.1 mg/mL), ArtinM (2.5 µg/mL), IL-4 (40 ng/mL), or medium alone (Medium). After 24 h of incubation, the cell culture supernatants were collected to measure the levels of TNF- α by ELISA (black bar). An additional 24 h of incubation a second stimulus was given as follows: (gray bar) fresh medium; (red bar) incubated with LPS (0.1 µg/mL), P3C4 (0.1 mg/mL), ArtinM (2.5 µg/mL), IL-4 (40 ng/mL), or Medium; (blue bar) IL-4 at a concentration of 10 ng/mL; (green bar) IL-4 at a concentration of 40 ng/mL; (yellow bar) IL-4 at a concentration of 80 ng/mL. After 24 h of culture, the quantification of levels of TNF- α was performed in cell culture supernatants by ELISA. The values are expressed in means ± SD and ∗p < 0.05, according to the Kruskal–Wallis test followed by Dunn’s multiple comparisons test. * Compared to the Medium. [file peerj-08-10295-s001.png]
